# Body mass index and lung cancer risk: a pooled analysis based on nested case-control studies from four cohort studies

**DOI:** 10.1186/s12885-018-4124-0

**Published:** 2018-02-23

**Authors:** Harinakshi Sanikini, Jian-Min Yuan, Lesley M. Butler, Woon-Puay Koh, Yu-Tang Gao, Annika Steffen, Mattias Johansson, Paolo Vineis, Gary E. Goodman, Matt J. Barnett, Rayjean J. Hung, Chu Chen, Isabelle Stücker

**Affiliations:** 1 Cancer and Environment Group, Center for Research in Epidemiology and Population Health (CESP), INSERM, Université Paris Saclay, Université Paris-Sud, Villejuif, France; 2 Division of Cancer Control and Population Sciences, University of Pittsburgh Cancer Institute, Pittsburgh, PA USA; 3 Department of Epidemiology, Graduate School of Public Health, University of Pittsburgh, Pittsburgh, PA USA; 4 Duke-NUS Medical School, Singapore, Singapore; 5 Saw Swee Hock School of Public Health, National University of Singapore, Singapore, Singapore; 6 Department of Epidemiology, Shanghai Cancer Institute, Shanghai, China; 7 Shanghai Jiaotong University School of Medicine, Shanghai, China; 8 German Institute of Human Nutrition Potsdam-Rehbrücke, Nuthetal, Germany; 9 International Agency for Research on Cancer, Lyon, France; 10 Department of Epidemiology and Biostatistics, the School of Public Health, Imperial College London, London, UK; 11 Division of Public Health Sciences, Fred Hutchinson Cancer Research Center, Seattle, WA USA; 12 Lunenfeld-Tanenbaum Research Institute, Sinai Health System, Toronto, Canada; 13 Program in Epidemiology, Division of Public Health Sciences, Fred Hutchinson Cancer Research Center, Seattle, WA USA

**Keywords:** Body mass index, Obesity, Overweight, Lung cancer

## Abstract

**Background:**

Obesity has been proposed as a potential protective factor against lung cancer. We examined the association between BMI and lung cancer risk in a pooled analysis based on nested case-control studies from four cohort studies.

**Methods:**

A case-control study was nested within four cohorts in USA, Europe, China and Singapore that included 4172 cases and 8471 control subjects. BMI at baseline was calculated as weight in kilograms divided by height in meters squared (kg/m^2^), and classified into 4 categories: underweight (BMI < 18.5), normal weight (18.5 ≤ BMI < 25), overweight (25 ≤ BMI < 30) and obese (≥30). Odds ratios (ORs) and 95% confidence intervals (CIs) for BMI-lung cancer associations were estimated using unconditional logistic regression, adjusting for potential confounders.

**Results:**

Considering all participants, and using normal weight as the reference group, a decreased risk of lung cancer was observed for those who were overweight (OR 0.77, 95% CI: 0.68–0.86) and obese (OR 0.69, 95% CI: 0.59–0.82). In the stratified analysis by smoking status, the decreased risk for lung cancer was observed among current, former and never smokers (P for interaction 0.002). The adjusted ORs for overweight and obese groups were 0.79 (95% CI: 0.68–0.92) and 0.75 (95% CI: 0.60–0.93) for current smokers, 0.70 (95% CI: 0.53–0.93) and 0.55 (95% CI: 0.37–0.80) for former smokers, 0.77 (95% CI: 0.59–0.99), and 0.71 (95% CI: 0.44–1.14) for never smokers, respectively. While no statistically significant association was observed for underweight subjects who were current smokers (OR 1.24, 95% CI: 0.98–1.58), former smokers (OR 0.27, 95% CI: 0.12–0.61) and never smokers (OR 0.83, 95% CI: 0.5.-1.28).

**Conclusion:**

The results of this study provide additional evidence that obesity is associated with a decreased risk of lung cancer. Further biological studies are needed to address this association.

**Electronic supplementary material:**

The online version of this article (10.1186/s12885-018-4124-0) contains supplementary material, which is available to authorized users.

## Background

Lung cancer is the most common cancer and the leading cause of cancer-related deaths worldwide, with an estimated 1.82 million lung cancer cases and 1.59 million deaths in 2012 [1]. Incidence and mortality rates for lung cancer are higher among men than women, with 1.2 million cases and 1 million deaths estimated in men and 580,000 cases and 490,000 deaths estimated in women in 2012 [2]. The incidence of lung cancer varies by age, sex, geographical location and histological type [3, 4]. These variations are mostly determined by differences in smoking patterns and exposures to other lung carcinogens [5–8]. Smoking, second-hand smoke, air pollution, asbestos, radon, and occupational exposure to chemical carcinogens are well-known risk factors for lung cancer [9–13]. Furthermore, a comprehensive review of epidemiological evidence revealed that low consumption of fruits and vegetables contribute to an increased risk of lung cancer [14, 15].

Obesity is linked to an increased risk of many cancers, including cancers of the breast (in post-menopausal women), endometrium, esophagus, gallbladder, kidney, colorectal, and pancreas [16]. By contrast, body mass index (BMI, a proxy measure of obesity) of ≥30 kg/m2, has been inversely associated with the risk of lung cancer in several case-control and cohort studies [17–27]. Besides, some of these studies have also shown that low BMI is associated with an increased risk of lung cancer [19, 20, 25, 28, 29]. Two recent meta-analyses have provided more evidence supporting that excess weight could significantly decrease the risk of lung cancer [30, 31]. There are some methodological issues in examining the association between BMI and lung cancer risk. Firstly, smoking is an established risk factor for lung cancer and is also associated with body weight, which may confound the relation between BMI and lung cancer [32, 33]. Smokers tend to be leaner than non-smokers; heavy smokers tend to have greater body weight than light smokers, which likely reflects an unhealthy lifestyle (for instance, poor diet and low level of physical activity) [32]. In fact, studies that restricted the analysis to never smokers, the association between BMI and lung cancer disappeared [34, 35]. Secondly, preclinical effects of lung cancer and associated weight loss may distort the association between BMI and lung cancer, which is often referred to as reverse causation [20, 36]. Studies that had a short follow-up or studies in which weight was reported shortly before cancer diagnosis are more prone to reverse causality. To our knowledge, few studies have attempted to tackle these methodological issues using Mendelian randomization approach [37–39]. However, this method has not been extended to evaluate non-linear associations. Apart from these, some epidemiological studies have failed to find the inverse association between BMI and lung cancer risk [40, 41]. In addition, histological types of lung cancer may exemplify largely divergent diseases with different etiologies, but studies examining the association between BMI and lung cancer by histological type are limited [24, 42, 43].

Hence, the aim of the present study was to examine the association between BMI and lung cancer risk in a pooled analysis based on nested case-control studies from four cohort studies in USA, Europe, China and Singapore. The large sample size of this nested study allowed us to assess the association by gender, smoking status and histological types of lung cancer.

## Methods

### Study population

This project was conducted under the framework of the International Lung Cancer Consortium (ILCCO). ILCCO was established in 2004 with the objective to pool equivalent data and maximize resource sharing and statistical power of epidemiological studies of lung cancer [44]. Four ILCCO studies are included in this pooled analysis. The collaborating cohorts have been described in detail previously [45–51]. These are the Carotene and Retinol Efficacy Trial (CARET), European Prospective Investigation into Cancer and Nutrition Study (EPIC), Shanghai Cohort Study (SCS), and Singapore Chinese Health Study (SCHS). A summary of selected characteristics of these cohorts is presented in Table 1.

**Table 1:** Characteristics of participating cohorts

Study	Location	Enrollment years	Baseline cohort	Age at enrollment	Follow-up mean years	Source of height and weight data	Cases/Controls (*N* = 4172/8471)	Matching
Carotene and Retinol Efficacy Trial	USA	1985–1994	18,314	45–69	11.5	Measured	787/1564	Age (± 4 years), sex, race, enrollment year (2-years intervals), baseline measures of smoking status (current or former), asbestos exposure (yes or no) and duration of follow up
European Prospective Investigation into Cancer and Nutrition	Europe	1992–2000	521,468	35–70	10.1	Mostly Measured, except for some EPIC centers ^a^	1242/2622	Age, sex, smoking status, and country of recruitment
Shanghai Cohort Study	China	1986–1989	18,244	45–64	15.8	Self-reported	965/1929	Age and sex
Singapore Chinese Health Study	Singapore	1993–1998	63,257	45–74	10.0	Self-reported	1178/2356	Age and sex

^a^Oxford cohort, Norwegian cohort and approximately two-thirds of the French cohort, height and weight were self-reported

### Cases ascertainment and data collection method

Cases included were all incident primary lung cancer (International Classification of Diseases-Oncology (ICD-O) 3rd edition and included all invasive cancers coded to C33–34). All histological types were included. Case ascertainment varied among studies but included linking participants to cancer registries, health insurance records, medical records, self-report, and next of kin reports. Most of the cases among studies were histologically confirmed.

In each study, two lung cancer-free controls were matched per case (controls were cancer-free at the time of diagnosis of the matched case). Mostly, controls were matched to cases on age (plus/minus 5 years) and sex. Some cohorts used more stringent matching on other variables (Table 1). In each study, two lung cancer-free controls were matched per case.

Data on demographics and possible confounders were collected among studies through a self-administered written questionnaire (EPIC and CARET) or in- person interviews (EPIC, SCS and SCHS). At recruitment, measurements of height and weight were taken for all the participants of the CARET study and for most of the EPIC cohort (Table 1). In the SCS and SCHS cohort and for some of the EPIC participants (mainly for Oxford cohort, Norwegian cohort and approximately two-thirds of the French cohort) height and weight at baseline were self-reported. A detailed description of data collection methods has been published previously by the individual studies [45–51]. From each study, baseline information on anthropometric measurements (height and weight), history of cigarette smoking, sex, age at enrollment and diagnosis, year of last observation/follow-up, and level of education was requested.

### Statistical analysis

Unconditional logistic regression models were used to estimate odds ratios (ORs) and 95% confidence intervals (CIs) for the association between BMI and lung cancer risk. BMI at baseline was calculated as weight in kilograms divided by the *square* of the height in meters (kg/m^2^) and classified into 4 categories according to the WHO international classification: underweight (BMI < 18.5), normal weight (18.5 ≤ BMI < 25), overweight (25 ≤ BMI < 30) and obese (≥30). Normal weight was used as the reference category. Pack-years of smoking were computed by using the formula: (number of years smoked x mean number of cigarettes smoked per day)/20. In cases, time elapsed was computed as the difference between the age at enrolment and diagnosis, whereas in controls, it was calculated as the difference between age at enrolment and last follow-up/observation.

All models were adjusted for sex, study center, age (< 45, 45–49, 50–54, 55–59, 60–64, 65–69, ≥70), time elapsed (< 2, 2–8, 9–14, 15–20, ≥20), pack-years of smoking (0, < 20, 20–29, 30–39, 40–49, and ≥50), and education level (none, primary school, middle/vocational, secondary school, postsecondary/technical and university). Subgroup analyses were performed for gender, smoking status and histologic types of lung cancer. Deviation of multiplicative interactions of BMI with sex and smoking status was explored by including an interaction term along with the main effect term in the adjusted model. The statistical significance of the interaction term was evaluated using likelihood ratio tests. To investigate possible reverse causation, sensitivity analysis was performed by excluding lung cancer cases diagnosed in the first 3 years of follow up. Additional, sensitivity analysis was also conducted by eliminating two studies (SCS and SCHS), where height and weight were self-reported. We tested for heterogeneity across studies using the Q and I^2^ statistic [52]. To graphically display odds ratios representing the dose-response association for BMI and lung cancer risk, we used the restrictive cubic spline (RCS) function with 4 knots (5, 10, 20, and 40 percentile) in a multivariate unconditional logistic regression model as described above. The selection of model (4 knots) was based on the lower Akaike Information Criteria (AIC). This analysis was performed using the RCS_Reg SAS Macro created by Desquilbet and Mariotti [53]. All analyses were performed using the SAS 9.3 software (SAS Institute, Cary, NC) and a *p*-value < 0.05 was considered as statistically significant.

## Results

The study included 4172 lung cancer cases and 8471 controls aged 35 to 74 years (Table 1). Baseline characteristics of participants are presented in Table 2. Of the 4172 lung cancer cases, 3043 were men and 1129 were women. Compared with controls, cases were slightly older, had a lower education level and higher prevalence of current smoking. The average age at lung cancer onset in cases was 68.0 years, and the average time elapsed from enrollment to diagnosis of lung cancer in cases was 8.3 years.

**Table 2:** Selected characteristics of participants

Characteristic	Cases (*n* = 4172) N (%)	Controls (*n* = 8471) N (%)	*P* value (X^2^)
Sex			0.37
Men	3043 (72.9)	6135 (72.4)	
Women	1129 (27.1)	2336 (27.6)	
Age			<.0001
< 45	76 (1.8)	197 (2.3)	
45–49	211 (5.1)	980 (11.6)	
50–54	602 (14.4)	1720 (20.3)	
55–59	1010 (24.2)	2165 (25.5)	
60–64	1216 (29.2)	2024 (23.9)	
65–69	731 (17.5)	1070 (12.6)	
≥ 70	326 (7.8)	324 (3.8)	
BMI (kg/m^2^) ^a^			<.0001
Underweight	250 (5.9)	408 (4.8)	
Normal weight	2150 (51.5)	4276 (50.5)	
Overweight	112 (26.7)	2746 (32.4)	
Obese	373 (8.9)	934 (11.0)	
Missing	287 (6.9)	107 (1.3)	
Education			<.0001
None	529 (12.7)	699 (8.3)	
Primary	1515 (36.3)	2518 (29.7)	
Middle/Vocational	851 (20.4)	2109 (24.9)	
Secondary	457 (11.0)	1060 (12.5)	
Postsecondary/Technical	312 (7.5)	665 (7.9)	
University	381 (9.1)	1164 (13.7)	
Unknown/not specified	26 (0.6)	51 (0.6)	
Missing	101 (2.4)	205 (2.4)	
Smoking status			<.0001
Never	571 (13.7)	3144 (37.1)	
Former	694 (16.6)	1735 (20.5)	
Current	2892 (69.3)	3556 (42.0)	
Missing ^b^	15 (0.36)	36 (0.4)	
Pack-years of smoking			<.0001
0	571 (13.7)	3144 (37.1)	
< 20	547 (13.1)	1562 (18.4)	
20–29	554 (13.3)	850 (10.0)	
30–39	691 (16.6)	891 (10.5)	
40–49	817 (19.6)	829 (9.8)	
≥ 50	831 (19.9)	892 (10.5)	
Missing	161 (3.9)	303 (3.6)	
Age at diagnosis of lung cancer, y			
Mean (SD)	68.0 (8.1)	–	
Median (range)	68.2 (38.1–91.0)		
Time elapsed, y			
Mean (SD)	8.3 (5.4) ^c^	13.3 (5.1) ^d^	
Median (range)	7.3 (0–27)	13.0 (0–28)	
Histological Type			
Adenocarcinoma	1182 (42.6)		
Squamous cell carcinoma	897 (32.6)		
Large cell carcinoma	221 (7.9)		
Small cell carcinoma	473 (17.1)		

^a^Underweight (BMI < 18.5), normal weight (18.5 ≤ BMI < 25), overweight (25 ≤ BMI < 30) and obese (BMI ≥ 30)

^b^Subjects who had missing cigarettes smoked per day and duration of smoking

^c^Period between enrollment and diagnosis

^d^Period between enrollment and last follow-up/observation

In the total participants, cases had slightly lower mean weight compared with controls (68.2 and 69.7 kg). Mean height was similar (1.67 m). Fifty-two percent of cases and 51% of controls had BMI in the normal range, 27% of cases and 32% of controls were overweight, and 9% of cases and 11% of controls were obese.

Table 3 displays adjusted ORs and 95% CIs for lung cancer according to baseline BMI categories. Considering all participants, and using normal weight as the reference group, a decreased risk of lung cancer was observed for those who were overweight (OR 0.77, 95% CI: 0.68–0.86) and obese (OR 0.69, 95% CI: 0.59–0.82) whereas no statistically significant association was observed for underweight subjects (OR 1.03, 95% CI: 0.84–1.25). When stratified by gender, the inverse association observed between BMI and lung cancer risk was similar for overweight and obese men (OR 0.71, 95% CI: 0.62–0.81 for overweight group; and OR 0.63, 95% CI: 0.52–0.78 for obese group); the association for women was slightly attenuated (OR 0.80, 95% CI: 0.63–1.02 for overweight group; and OR 0.70, 95% CI: 0.51–0.97 for obese group) (Table 3).

**Table 3:** Adjusted odds ratio of lung cancer according to BMI categories

	Men and Women			Men			Women		
	Cases	Controls	Adjusted	Cases	Controls	Adjusted	Cases	Controls	Adjusted
BMI (kg/m^2^) ^a^	(*N* = 4172)	(*N* = 8471)	OR (95% CI) ^b^	(*N* = 3043)	(*N* = 6135)	OR (95% CI) ^c^	(*N* = 1129)	(*N* = 2336)	OR (95% CI) ^c^
Underweight	250	408	1.03 (0.84–1.25)	196	310	1.06 (0.85–1.32)	54	98	0.85 (0.52–1.39)
Normal weight	2150	4276	Reference	1607	3155	Reference	543	1121	Reference
Overweight	1112	2746	0.77 (0.68–0.86)	810	2004	0.71 (0.62–0.81)	302	742	0.80 (0.63–1.02)
Obese	373	934	0.69 (0.59–0.82)	256	628	0.63 (0.52–0.78)	117	306	0.70 (0.51–0.97)
Missing	287	107	–	174	38	–	113	69	–

^a^Underweight (BMI < 18.5), normal weight (18.5 ≤ BMI < 25), overweight (25 ≤ BMI < 30) and obese (BMI ≥ 30)

^b^Adjusted for age, gender, study center, time elapsed, pack-years of smoking and education level

^c^Adjusted for age, study center, time elapsed, pack-years of smoking and education level

To further investigate the association between BMI and lung cancer risk among subgroups, we stratified the analyses by smoking status (Table 4). In both genders combined, the decreased risk for lung cancer was observed among current, former, and never smokers (P for interaction 0.002). The adjusted ORs for overweight and obese groups were 0.79 (95% CI: 0.68–0.92) and 0.75 (95% CI: 0.60–0.93) for current smokers, 0.70 (95% CI: 0.53–0.93) and 0.55 (95% CI: 0.37–0.80) for former smokers, 0.77 (95% CI: 0.59–0.99), and 0.71 (95% CI: 0.44–1.14) for never smokers, respectively. When separate analysis was performed by gender, the decreased risk for lung cancer was observed among both former and current male and female smokers but it did not reach statistical significance among female smokers, which could be explained by few number of female cases (Table 4).

**Table 4:** Adjusted odds ratio of lung cancer by smoking status according to BMI categories

BMI (kg/m^2^) ^a^		Men and Women			Men			Women	
	Cases	Controls	Adjusted OR (95% CI) ^b^	Cases	Controls	Adjusted OR (95% CI) ^c^	Cases	Controls	Adjusted OR (95% CI) ^c^
*Never Smokers*									
Underweight	33	161	0.83 (0.53–1.28)	15	97	0.88 (0.49–1.60)	18	64	0.84 (0.42–1.68)
Normal weight	334	1794	Reference	169	1159	Reference	165	635	Reference
Overweight	117	916	0.77 (0.59–0.99)	46	540	0.74 (0.51–1.06)	71	376	0.75 (0.51–1.11)
Obese	29	233	0.71 (0.44–1.14)	5	110	0.55 (0.22–1.40)	24	123	0.73 (0.40–1.36)
Missing	58	40	–	19	15	–	39	25	–
*Former Smokers*									
Underweight	10	58	0.27 (0.12–0.61)	8	51	0.26 (0.11–0.61)	2	7	0.27 (0.02–3.69)
Normal weight	262	609	Reference	203	477	Reference	59	132	Reference
Overweight	272	729	0.70 (0.53–0.93)	224	599	0.66 (0.48–0.91)	55	130	0.77 (0.36–1.65)
Obese	105	297	0.55 (0.37–0.80)	83	230	0.51 (0.33–0.79)	22	67	0.67 (0.27–1.67)
Missing	38	42		26	15	–	–	27	–
*Current Smokers*									
Underweight	207	189	1.24 (0.98–1.58)	173	162	1.26 (0.98–1.63)	34	27	0.82 (0.38–1.76)
Normal weight	1549	1860	Reference	1234	1511	Reference	315	349	Reference
Overweight	710	1085	0.79 (0.68–0.92)	537	853	0.75 (0.63–0.88)	173	232	0.87 (0.62–1.23)
Obese	237	400	0.75 (0.60–0.93)	167	286	0.72 (0.55–0.92)	70	114	0.73 (0.46–1.15)
Missing	189	22	–	127	6	–	62	16	–

^a^Underweight (BMI < 18.5), normal weight (18.5 ≤ BMI < 25), overweight (25 ≤ BMI < 30) and obese (BMI ≥ 30)

^b^Adjusted for age, gender, study center, time elapsed, pack-years of smoking (except for never smokers) and education level

^c^Adjusted for age, study center, time elapsed, pack-years of smoking (except for never smokers) and education level

**Fig. 1:**
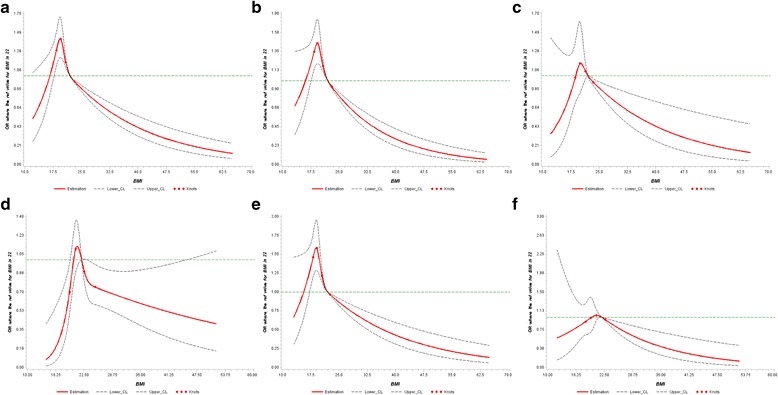
Adjusted dose-response association between BMI and risk of lung cancer: (**a**) All participants (**b**) Men (**c**) Women (**d**) Former smokers (**e**) Current smokers (**f**) Never smokers. BMI was coded using an RCS function with four knots arbitrarily located at the 5th, 10th, 20th and 40th percentiles. The y-axis represents the adjusted odds ratio for lung cancer risk for any value of BMI compared to individuals with a BMI of 22.0 kg/m^2^ (median value of BMI). Dashed lines are 95% CI. Knots are represented by dots

We performed RCS regression to describe the nonlinear dose-response association between BMI and risk of lung cancer (Fig. 1). In all the participants, we found a significant nonlinear dose-response association between BMI and risk of lung cancer (P _nonlinearity_ 0.001; Fig. 1a). After stratifying by gender, the evidence of a nonlinear association was observed in men (P _nonlinearity_ 0.009; Fig. 1b) but not in women (P _nonlinearity_ 0.11; Fig. 1c). After stratifying by smoking status, the nonlinear association was observed in former and current smokers (P _nonlinearity_ 0.006; Fig. 1d,e respectively) but not in never smokers (P _nonlinearity_ 0.14; Fig. 1f).

We also examined the association between BMI and risk of histological types of lung cancer (Table 5). When we stratified the analysis by histological types, the reduction in risk was observed for all histological types but it was statistically significant for adenocarcinoma and large cell carcinoma. The adjusted ORs for overweight and obese groups were 0.74 (95% CI: 0.62–0.87) and 0.65 (95% CI: 0.50–0.85) for adenocarcinoma, 0.69 (95% CI: 0.48–0.99) and 0.49 (95% CI: 0.26–0.92) for large cell carcinoma, respectively.

**Table 5:** Adjusted odds ratio of lung cancer by histological type according to BMI categories

BMI (kg/m^2^) ^a^	Cases	Controls	Adjusted OR (95%CI) ^b^
*Adenocarcinoma*			
Underweight	84	408	1.17 (0.89–1.54)
Normal weight	623	4288	Reference
Overweight	290	2752	0.74 (0.62–0.87)
Obese	90	936	0.65 (0.50–0.85)
Missing	95	106	–
*Squamous cell carcinoma*			
Underweight	56	408	1.06 (0.76–1.47)
Normal weight	445	4288	Reference
Overweight	259	2752	0.89 (0.74–1.08)
Obese	85	936	0.77 (0.57–1.03)
Missing	52	106	–
*Large cell carcinoma*			
Underweight	15	408	0.98 (0.55–1.76)
Normal weight	117	4288	Reference
Overweight	54	2752	0.69 (0.48–0.99)
Obese	13	936	0.49 (0.26–0.92)
Missing	22	106	–
*Small cell carcinoma*			
Underweight	14	408	0.62 (0.35–1.11)
Normal weight	208	4288	Reference
Overweight	155	2752	0.90 (0.70–1.15)
Obese	57	936	0.79 (0.56–1.12)
Missing	39	106	–

^a^Underweight (BMI < 18.5), normal weight (18.5 ≤ BMI < 25), overweight (25 ≤ BMI < 30) and obese (BMI ≥ 30)

^b^Adjusted for age, gender, study center, time elapsed, pack-years of smoking and education level

The risk estimates did not change substantially in the sensitivity analysis after exclusion of lung cancer cases diagnosed in the first 3 years of follow-up (Additional file 1: Table S1). In addition, analyses using measured BMI, which included two studies (CARET and EPIC) yielded similar results (data not shown).There was mild heterogeneity between cohorts for the BMI-lung cancer results (*P* value = 0.12, I^2^ = 50%) (Additional file 2: Figure S1).

## Discussion

In this pooled analysis of cohorts involving 12,643 subjects (4172 lung cancer cases and 8471 controls), we found a statistically significant inverse, dose-dependent association between BMI and lung cancer risk. This inverse association was present in current, former and never smokers and the effect was more evident for the subjects with a BMI of > 30 (kg/m^2^).

Our study found that being overweight or obese is associated with a decreased risk of lung cancer. This is consistent with a recent meta-analysis including 31 studies (20 cohorts, 11 case-control). The pooled relative risks in this study were 0.74 (95% CI: 0.68–0.80) for overweight (BMI 25–29.9 kg/m^2^) and 0.71 (95% CI: 0.68–0.80) for obese (BMI ≥30 kg/m^2^), compared with normal weight (BMI 18.5–24.9 kg/m^2^) [30]. More recently Duan et al. conducted a dose-response meta-analysis, which included 29 cohort studies and found evidence of a non-linear association between BMI and lung cancer risk (P _nonlinearity_ < 0.001) [31]. Compared with individuals with a BMI of 18.5–24.9 kg/m^2^, the summary relative risks for those with a BMI of 30 kg/m^2^ and BMI 35 kg/m^2^ were 0.91 (95% CI: 0.85–0.98) and 0.81 (95% CI: 0.72–0.91), respectively [31]. A cohort study conducted in the UK, which was not included in this meta-analysis, also showed an inverse association between higher BMI and lung cancer risk [54].

In gender-stratified analysis, we observed similar results in both men and women. The findings of previous meta-analyses also indicated no gender differences in the association between BMI and lung cancer risk [30, 31].

Given that smoking is the most important risk factor for lung cancer and associated with body weight, we stratified our analyses by smoking status and found a significant inverse association between BMI and lung cancer risk among current, former and never smokers. This is in line with results of previous meta-analysis [31]. In this meta-analysis, the pooled RRs for overweight and obese groups were 0.91 (95% CI: 0.85–0.98) and 0.77 (95% CI: 0.69–0.85) for ex-smokers, 0.79 (95% CI: 0.71–0.87), 0.72 (95% CI: 0.66–0.78) for current smokers and 0.86 (95% CI: 0.78–0.94) and 0.86 (95% CI: 0.75–0.98) for non-smokers respectively [31].

In our study, stratification by histological subtype showed that overweight and obese was significantly inversely associated with risk of adenocarcinoma and large cell carcinoma. The results of stratified analysis in the previous meta-analysis reported a lower risk for adenocarcinoma and squamous cell carcinoma [30, 31].

Our study found no association between being underweight and risk of lung cancer. However, stratification by smoking status showed a non-significant increased risk for lung cancer in current smokers who were underweight (OR 1.24, 95% CI: 0.98–1.58). Results of a recent meta-analysis reported a significant positive association between low BMI and lung cancer risk (pooled RR 1.24; 95% Cl: 1.20–1.27; for underweight vs. normal weight) [31]. But, this association was confined to current smokers (RR 1.31, 95% CI: 1.10–1.57) and no statistically significant association was found in ex-smokers and never smokers (RR 1.40, 95% CI: 0.82–2.36 and RR 1.18, 95% CI: 0.90–1.54, respectively) [31].

A few biological mechanisms support the plausibility for the inverse association between BMI and lung cancer risk. Environmental Geno toxicants like polycyclic aromatic hydrocarbons (PAHs) that derived from smoking and occupational exposure, are known to cause DNA damage that results in a dose-dependent risk of lung cancer [55]. Among PAHs, benzo-α-pyrene is the most widely studied element, and its ability to induce lung tumors upon inhalation is well recognized [56]. Interestingly, studies have found inverse associations between BMI and benzo-α-pyrene DNA adduct levels among smokers, suggesting that increased body fat impacts adduct levels, possibly by affecting the distribution of the carcinogen [57, 58]. In addition, inverse associations have been reported between BMI and levels of urinary 8-hydroxydeoxyguanosine (8-OHdG), a marker of oxidative DNA damage in smokers [59, 60]. Brennan et al. reported that FTO genotype, a genetic marker of obesity which is related to increased BMI, was associated with a decreased risk of lung cancer [37]. On the other hand, two recent Mendelian randomization (MR) analyses for BMI and lung cancer, showed increased BMI was positively associated with lung cancer risk [38, 39]. However, the MR assumptions can be confounded by the potential pleiotropic effects of genetic variants associated with both BMI and smoking behavior [38, 39]. Recently Dik et al. conducted a large-scale genome-wide analysis of the association between BMI and DNA methylation and found increased BMI is associated with increased methylation at the HIF3A locus in blood and in adipose tissue [61]. HIF3A is an element of the hypoxia-inducible transcription factor (HIF) that controls a wide variety of cellular and physiological responses to reduced oxygen concentrations by controlling the expression of several target genes [62]. Studies have observed that HIF3A can regulate many genes associated with angiogenesis, in addition to cell survival and apoptosis [63, 64]. These observations suggest that HIF3A may play a role in lung carcinogenesis [60]. However, further molecular-epidemiological studies are needed in exploring the underlying carcinogenic mechanisms associating BMI with lung cancer risk.

The major strengths of this study include its prospective cohort-based nested case-control design, large sample size, and available information on potential confounders. As the study population was largely a nested sample from different prospective cohort studies and BMI was measured before lung cancer diagnosis, hence the possibility of selection and recall bias is minimal. In addition, we were able to perform the analyses by gender, smoking status and histological types. Our study also has some limitations. First, the use of some self-reported exposure information. However, separate analyses using measured BMI yielded similar results. In addition, previous studies have noted that even though self-reported height tends to be overestimated and weight tends to be underestimated, the self-reported values are highly correlated with the measured values [65–67]. Second, our analyses were based on self-reported cigarette smoking at baseline, and information on change in smoking habits during follow-up was not available from studies; if smoking habits varied over time, this could have had some effect on the results. However, we observed similar patterns for former and current smokers; hence it is unlikely to alter the results if current smokers became former smokers during follow-up. Third, adjustment for pack-years of smoking may not adequately control for confounding effect of cigarette smoking, thus residual confounding by smoking may still exist. Last, there was a wide range of time elapsed between BMI measurement (collected at baseline) and the date of diagnosis. However, sensitivity analysis examining this time elapsed by excluding the first 3 years of follow-up did not change risk estimates substantially. Hence, it argues against an effect of preclinical disease-related changes in anthropometric measures (reverse causation).

## Conclusions

In conclusion, the results of this study provide additional evidence that obesity is associated with a decreased risk of lung cancer. Further biological studies are needed to address this association.

## Additional files

Additional file 1: **Table S1.** Adjusted odds ratio of lung cancer according to BMI categories after excluding first 3 years of cases. (DOCX 15 kb)

Additional file 2: **Figure S1.** Forest plot for the association between BMI and lung cancer risk. (DOCX 51 kb)

## Abbreviations

BMI: Body mass index
CARET: Carotene and Retinol Efficacy Trial
CI: Confidence interval
EPIC: European Prospective Investigation into Cancer and Nutrition Study
OR: Odds ratio
SCHS: Singapore Chinese Health Study
SCS: Shanghai Cohort Study
