# Supplementary material for: Body mass index and lung cancer risk: a pooled analysis based on nested case-control studies from four cohort studies
Source: BMC Cancer. 2018 Feb 23;18:220. doi: 10.1186/s12885-018-4124-0 (PMC5824613; doi:10.1186/s12885-018-4124-0)
Supplement: Supplementary file 1 — Table S1. Adjusted odds ratio of lung cancer according to BMI categories after excluding first 3 years of cases. (DOCX 15 kb) [file 12885_2018_4124_MOESM1_ESM.docx]

Supplementary Table 1. Adjusted odds ratio of lung cancer according to BMI categories after excluding first 3 years of cases

|  |  | Men and Women | |  |  | Men |  |  |  | Women |  |
| --- | --- | --- | --- | --- | --- | --- | --- | --- | --- | --- | --- |
|  | Cases | Controls | Adjusted |  | Cases | Controls | Adjusted |  | Cases | Controls | Adjusted |
| BMI (kg/m^2^) ^1^ | (N=3,439) | (N=8,471) | OR (95% CI) ^2^ |  | (N=2,533) | (N=6,135) | OR (95% CI) ^3^ |  | (N=906) | (N=2,336) | OR (95% CI) ^3^ |
| Underweight | 198 | 408 | 1.00 (0.81-1.24) |  | 157 | 310 | 1.02 (0.81-1.29) |  | 41 | 98 | 0.90 (0.54-1.49) |
| Normal weight | 1789 | 4276 | Reference |  | 1371 | 3155 | Reference |  | 418 | 1121 | Reference |
| Overweight | 911 | 2746 | 0.76 (0.67-0.85) |  | 656 | 2004 | 0.69 (0.60-0.79) |  | 255 | 742 | 0.82 (0.65-1.05) |
| Obese | 324 | 934 | 0.70 (0.59-0.83) |  | 218 | 628 | 0.63 (0.51-0.77) |  | 106 | 306 | 0.72 (0.52-1.00) |
| Missing | 217 | 107 | - |  | 131 | 38 | - |  | 86 | 69 | - |

^1^ Underweight (BMI<18.5), normal weight (18.5≤BMI<25), overweight (25≤BMI<30) and obese (BMI ≥ 30)

^2^ Adjusted for age, gender, study centre, time elapsed, pack-years of smoking and education level

^3^ Adjusted for age, study centre, time elapsed, pack-years of smoking and education level
