# Supplementary figures and images for: Body mass index and lung cancer risk: a pooled analysis based on nested case-control studies from four cohort studies
Source: BMC Cancer. 2018 Feb 23;18:220. doi: 10.1186/s12885-018-4124-0 (PMC5824613; doi:10.1186/s12885-018-4124-0)

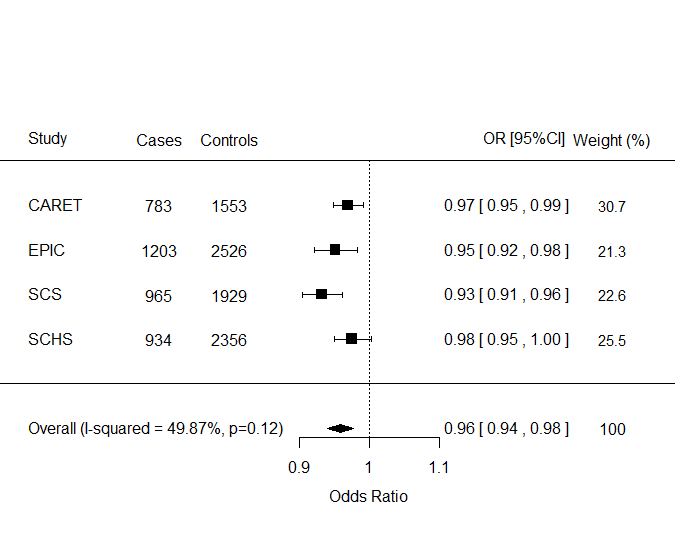

Supplement: Supplementary file 2 — Figure S1. Forest plot for the association between BMI and lung cancer risk. (DOCX 51 kb) [file 12885_2018_4124_MOESM2_ESM.docx]
